# The Potential of Avian H1N1 Influenza A Viruses to Replicate and Cause Disease in Mammalian Models

**DOI:** 10.1371/journal.pone.0041609

**Published:** 2012-07-25

**Authors:** Zeynep A. Koçer, Scott Krauss, David E. Stallknecht, Jerold E. Rehg, Robert G. Webster

**Affiliations:** 1 Department of Infectious Diseases, Division of Virology, St Jude Children’s Research Hospital, Memphis, Tennessee, United States of America; 2 Southeastern Cooperative Wildlife Disease Study, Department of Population Health, College of Veterinary Medicine, University of Georgia, Athens, Georgia, United States of America; 3 Department of Pathology, St Jude Children’s Research Hospital, Memphis, Tennessee, United States of America; The University of Hong Kong, China

## Abstract

H1N1 viruses in which all gene segments are of avian origin are the most frequent cause of influenza pandemics in humans; therefore, we examined the disease-causing potential of 31 avian H1N1 isolates of American lineage in DBA/2J mice. Thirty of 31 isolates were very virulent, causing respiratory tract infection; 22 of 31 resulted in fecal shedding; and 10 of 31 were as pathogenic as the pandemic 2009 H1N1 viruses. Preliminary studies in BALB/cJ mice and ferrets showed that 1 of 4 isolates tested was more pathogenic than the pandemic 2009 H1N1 viruses in BALB/cJ mice, and 1 of 2 strains transmitted both by direct and respiratory-droplet contact in ferrets. Preliminary studies of other avian subtypes (H2, H3, H4, H6, H10, H12) in DBA/2J mice showed lower pathogenicity than the avian H1N1 viruses. These findings suggest that avian H1N1 influenza viruses are unique among influenza A viruses in their potential to infect mammals.

## Introduction

Of the 5 influenza pandemics that have occurred in humans during the past century, 3 were caused by H1N1 viruses (1918 Spanish influenza, 1977 Russian influenza, and 2009 pandemic influenza). The finding that elderly people were protected against infection during the 1918 pandemic, while young people died from the disease raises speculation that H1N1 influenza viruses had circulated before 1918. Genetic analysis of human pandemic H1N1 and swine H1N1 viruses supports the contention that they are all derived from an avian origin [1]–[4]. It has been proposed that the 1918 Spanish influenza evolved independently, after it was introduced to swine and humans around the same time (1905–1915) [4], [5]. However, a recent phylogenetic study suggests that the 1918 virus was a reassortant between the circulating human strain and some other mammalian viruses of avian origin [6]. Classical swine influenza is considered to have spread from humans prior to its detection in the early 1930 s [7]. In European pigs, classical swine influenza was replaced by a wholly avian H1N1 virus prior to 1979 [8], and that virus established the European swine lineage.

H1N1 was the main cause of seasonal influenza until the mid-1950 s. It then disappeared for almost 20 years, before reappearing in 1977 (Russian influenza) with a molecular structure identical to that of a virus that had circulated during the late 1940 s, causing a pandemic mainly among young people. After more than 30 years, H1N1 re-emerged again in 2009, causing a pandemic that mostly affected young people. Molecular studies of the 2009 H1N1 virus indicate that it was derived from a combination of gene segments of avian, swine, and human origins [9]. The gene segments of swine origin were from North American and Eurasian swine lineages, and most importantly, all of them were ultimately derived from an avian origin [10].

The main question addressed in this study is whether avian H1N1 viruses have the unique ability to transfer to mammals and whether the mouse is a suitable “first mammal” model in which to identify those isolates that have the potential to transmit and cause disease in mammals. In a broader perspective, understanding the potential pathogenicity of avian H1N1 viruses in a first mammal model would be the first step toward establishing a risk-assessment and a risk-management program for zoonotic transmission in mammals. Among the commercially available and commonly used mouse strains of different genetic backgrounds, DBA/2J mice are the most susceptible to infection with high- or low-pathogenic influenza viruses [11]–[13].

In our North American influenza repository from migratory waterfowl collected over 35 years, about 6% of the isolates are H1N1 viruses (about 5% were isolated from Anseriformes, and about 0.2% were isolated from Charadriiformes). Here, we examine the pathogenicity of 31 representative avian H1N1 isolates from our repository and the clinical symptoms caused by these viruses in DBA/2J mice. Also, we present preliminary comparative studies in BALB/cJ mice and ferrets. Finally, we report preliminary pathogenicity studies of H2, H3, H4, H6, H10, and H12 in DBA/2J mice. The avian H1N1 viruses were surprisingly lethal in DBA/2J mice, and some strains were pathogenic in the BALB/cJ mice and ferrets.

## Results

### Pathogenicity of Avian H1N1 Viruses in DBA/2J Mice

To understand the ability of avian H1N1 influenza A viruses to replicate and cause disease in mammals, we screened 31 H1N1 isolates from our repository of viruses obtained from wild aquatic birds in DBA/2J mice. We also compared the pathogenicity of those isolates with that of 2 pandemic 2009 H1N1 isolates.

Thirty of 31 (97%) viruses caused 100% fatality in DBA/2J mice at 10^6^ EID_50_ (50% egg infectious dose), which was used as an end result, and 1 isolate (A/green-winged teal/LA/Sg-00090/2007) caused 20% mortality. On the basis of the mortality and morbidity caused by these viruses, we categorized them into 4 main groups and assigned a pathogenicity index (PI) value to each. PI values were calculated using percent survival by time and percent weight loss by time (Table 1).

**Table 1:** Percent mortality and pathogenicity in DBA/2J mice infected with avian H1N1 virus isolates or pandemic H1N1 viruses (at 10^6^ EID_50_).

Influenza A virus isolate	Subtype	Mortality (%)	Survival score^a^	Weight lossscore^b^	Total pathogenicity score^c^	Pathogenicity Index (PI)^d^
A/mallard/ALB/119/1998	H1N1	100	0.286	0.061	0.347	4
A/mallard/MN/AI07-3100/2007	H1N1	100	0.286	0.061	0.347	4
A/shorebird/DE/300/2009	H1N1	100	0.309	0.073	0.382	4
A/mallard/ALB/201/1998	H1N1	100	0.320	0.074	0.394	4
A/pintail/ALB/210/2002	H1N1	100	0.343	0.072	0.415	4
A/mallard/OH/4809-9/2008	H1N1	100	0.343	0.073	0.416	4
A/mallard/MN/AI07-3136/2007	H1N1	100	0.343	0.077	0.419	4
A/mallard/MN/AI07-3127/2007	H1N1	100	0.343	0.078	0.421	4
A/shorebird/DE/324/2009	H1N1	100	0.377	0.087	0.464	4
A/mallard/ALB/88/2004	H1N1	100	0.366	0.107	0.472	4
A/redheaded duck/MN/Sg-00123/2007	H1N1	100	0.400	0.084	0.484	3
A/pintail/ALB/68/2005	H1N1	100	0.400	0.085	0.485	3
A/pintail/ALB/69/2005	H1N1	100	0.400	0.085	0.485	3
A/mallard/MN/Sg-00121/2007	H1N1	100	0.400	0.086	0.486	3
A/northern shoveler/MN/Sg-00651/2008	H1N1	100	0.400	0.086	0.486	3
A/gull/DE/428/2009	H1N1	100	0.400	0.088	0.488	3
A/northern shoveler/MN/Sg-00655/2008	H1N1	100	0.400	0.088	0.488	3
A/mallard/ALB/267/1996	H1N1	100	0.400	0.091	0.491	3
A/shorebird/DE/170/2009	H1N1	100	0.400	0.092	0.492	3
A/northern shoveler/MO/466554-14/2007	H1N1	100	0.411	0.096	0.507	3
A/mallard/MN/AI07-3018/2007	H1N1	100	0.423	0.100	0.523	3
A/blue-winged teal/LA/B228/1986	H1N1	100	0.457	0.100	0.557	3
A/mallard/TN/11464/1985	H1N1	100	0.457	0.100	0.557	3
A/mallard/MN/AI07-3140/2007	H1N1	100	0.457	0.102	0.559	3
A/canvasback/ALB/276/2005	H1N1	100	0.457	0.102	0.559	3
A/blue-winged teal/ALB/212/1984	H1N1	100	0.457	0.104	0.561	3
A/mallard/MN/Sg-00628/2008	H1N1	100	0.457	0.106	0.563	3
A/shorebird/DE/318/2009	H1N1	100	0.514	0.119	0.633	2
A/mallard/MN/Sg-00627/2008	H1N1	100	0.571	0.126	0.697	2
A/shorebird/DE/274/2009	H1N1	100	0.571	0.155	0.726	2
A/green-winged teal/LA/Sg-00090/2007	H1N1	20	0.766	0.199	0.965	1
A/CA/04/2009	(pdm H1N1)	100	0.274	0.063	0.337	4
A/TN/1-560/2009	(pdm H1N1)	100	0.331	0.096	0.428	4

a The survival score was calculated as 0.8× (survival AUC/maximum AUC).

b The weight loss score was calculated as 0.2× (weight loss AUC/maximum AUC).

c The total pathogenicity score is the sum of the survival and weight loss scores.

d Pathogenicity indexes were classified as follows: 4, most pathogenic; 3, moderately pathogenic; 2, low pathogenic; and 1, least pathogenic.

**Figure 1:**
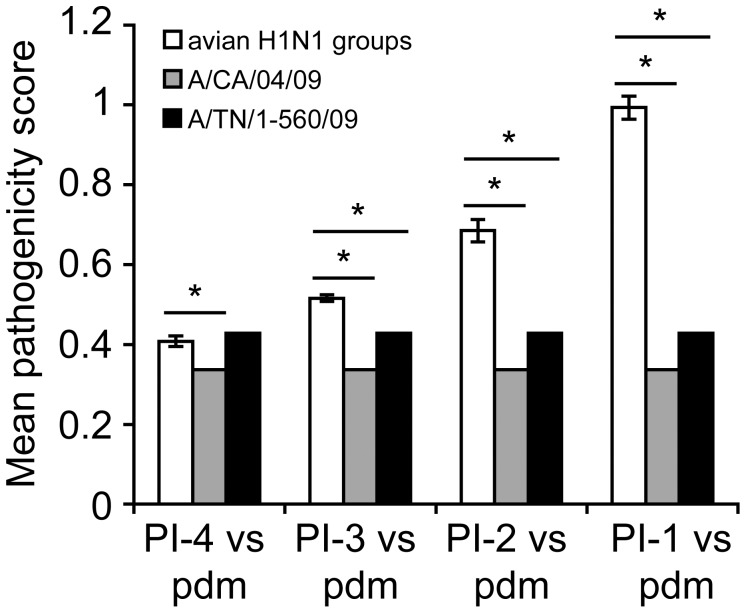
Comparison of the mean pathogenicity scores of each pathogenicity index (PI) category. Each avian H1N1 virus and the 2009 pandemic strains were assigned to a PI category based on their total pathogenicity score in DBA/2J mice. The PI-4 category includes the most pathogenic isolates; PI-3, moderately pathogenic isolates; PI-2, low pathogenic isolates; and PI-1, least pathogenic isolates. (*P<0.05).

All 10 viruses in the PI-4 category (the most pathogenic group) caused death or the maximum-acceptable weight loss sooner than the less pathogenic strains. The viruses with lower pathogenicity caused infections that progressed slowly, and mortality was delayed. The two 2009 pandemic H1N1 strains (A/CA/04/2009 and A/TN/1-560/2009) also resulted in 100% mortality in DBA/2J mice at the same infectious dose. Their pathogenicity scores were 0.337 and 0.428, respectively; thus, they were placed in the PI-4 category. The mean pathogenicity scores for each group were compared by one-way ANOVA, and the difference between them was statistically significant at p<0.05. When the mean pathogenicity score for each group was compared with that of each pandemic strain, statistically significant differences were observed for each group against A/CA/04/2009 (Figure 1). However, the mean pathogenicity score for viruses in the PI-4 category did not differ from that of the A/TN/1-560/2009 pandemic strain. The mean pathogenicity scores from the other 3 groups significantly differed from that of A/TN/1-560/2009. Thus, the majority of avian H1N1 viruses in the PI-4 group (8/10) have equal or lower pathogenicity scores than A/TN/1-560/2009, which means that avian H1N1 viruses might be more pathogenic than the recent 2009 pandemic strains.

Significant differences in clinical signs were observed, even across viruses within the same PI category. Some groups of mice showed scruffy fur, hunched appearance, less bright-alert response, and sudden weight loss as early as 3 days postinfection (dpi), while symptoms were delayed in the other groups. Therefore, in addition to survival and weight loss data, we determined the mouse 50% lethal dose (MLD_50_) for 9 selected viruses (7 viruses from the PI-4 category, 1 from the PI-3 category, and 1 from the PI-2 category) in terms of EID_50_ to distinguish within and between the pathogenicity categories. All 7 viruses from the PI-4 category killed 50% of mice at various lower infectious doses (10^1.68^ EID_50_/mL to 10^3.38^ EID_50_/mL) (Table 2). This result indicates the distinctive differences among the viruses, even in the same PI category. As expected, the MLD_50_ increased as pathogenicity decreased (10^4.0^ EID_50_/mL for PI-3 viruses and 10^5.22^ EID_50_/mL for PI-2 viruses).

**Table 2:** Fifty percent mouse lethal dose (MLD_50_) is inversely related to the pathogenicity index of avian H1N1 isolates.

H1N1 influenza A virus isolate	MLD_50_ (EID_50_ per mL)	Pathogenicity index (PI)
A/mallard/ALB/119/1998	10^1.68^	4
A/mallard/ALB/201/1998	10^1.75^	4
A/shorebird/DE/300/2009	10^2.17^	4
A/mallard/MN/AI07-3136/2007	10^2.63^	4
A/mallard/ALB/88/2004	10^2.69^	4
A/mallard/MN/AI07-3100/2007	10^2.83^	4
A/shorebird/DE/324/2009	10^3.38^	4
A/blue-winged teal/LA/B228/1986	10^4.0^	3
A/mallard/MN/Sg-00627/2008	10^5.22^	2

### Viral Titers in the Organs and Feces of DBA/2J Mice

To examine whether systemic infection occurs in mice after inoculation with avian H1N1 viruses, we collected several organs (i.e., spleen, liver, heart, brain, lungs, and intestines) from randomly selected groups of mice and examined the viral titers of those tissues to see if viral replication occurred. Viral titers were not detected in the spleen, liver, heart, or brain. Therefore, the virus did not spread systemically. Lungs and intestines were collected at various time points (5–10 dpi) from 27 groups of DBA/2J mice infected with avian H1N1 viruses to determine the viral titers. The average viral titers in the lungs of each group ranged from 10^4.94^/mL to 10^7.25^/mL EID_50_ (Table 3). The lowest lung titer (10^4.94^/mL EID_50_) was detected 10 dpi in a group of mice infected with A/mallard/MN/Sg-00627/2008. In comparison, the average viral titers in the lungs of DBA/2J mice at 5 dpi were 10^6.25^/mL EID_50_ in mice infected with A/CA/04/2009 and 10^6.5^/mL EID_50_ in those infected with A/TN/1-560/2009. Overall, viral titers in the lungs were not correlated with the PI values. In addition, severe lesions were observed in all of the lung lobes.

Viral titers in the intestines were determined in 24 groups of mice infected with different avian H1N1 viruses. Average titers ranged from less than 10^1^/mL to 10^4.25^/mL EID_50_. Although no viral titer was detected in 9 of the 24 groups, modest viral titers were detected in 6 groups (10^2.25^/mL-10^4.25^/mL EID_50_). The highest titer was detected 5 dpi in a mouse infected with A/shorebird/DE/300/2009, and the mean viral titer for the group of mice challenged with that virus was 10^3.38^/mL EID_50_. No viral titer was detected in the intestines of mice infected with either pandemic strain.

**Table 3:** Viral titers in the lungs of DBA/2J mice infected with avian H1N1 virus isolates from different pathogenicity levels.^a^

Influenza A virus isolate	Subtype	PathogenicityIndex (PI)	Days post infection	Average viral titer (in log_10_ EID_50_)
A/mallard/ALB/119/1998	H1N1	4	5	6.50
A/mallard/MN/AI07-3100/2007	H1N1	4	5	5.88
A/shorebird/DE/300/2009	H1N1	4	5	6.25
A/mallard/ALB/201/1998	H1N1	4	5	6.88
A/pintail/ALB/210/2002	H1N1	4	6	6.63
A/mallard/OH/4809-9/2008	H1N1	4	6	5.63
A/mallard/MN/AI07-3136/2007	H1N1	4	6	6.00
A/mallard/MN/AI07-3127/2007	H1N1	4	6	5.25
A/shorebird/DE/324/2009	H1N1	4	6	6.25
A/mallard/ALB/88/2004	H1N1	4	6	6.13
A/redheaded duck/MN/Sg-00123/2007	H1N1	3	7	5.50
A/pintail/ALB/68/2005	H1N1	3		ND
A/pintail/ALB/69/2005	H1N1	3		ND
A/mallard/MN/Sg-00121/2007	H1N1	3	7	5.50
A/northern shoveler/MN/Sg-00651/2008	H1N1	3	7	6.88
A/gull/DE/428/2009	H1N1	3		ND
A/northern shoveler/MN/Sg-00655/2008	H1N1	3		ND
A/mallard/ALB/267/1996	H1N1	3	7	6.63
A/shorebird/DE/170/2009	H1N1	3	7	6.63
A/northern shoveler/MO/466554-14/2007	H1N1	3	6	6.00
A/mallard/MN/AI07-3018/2007	H1N1	3	8	5.75
A/blue-winged teal/LA/B228/1986	H1N1	3	8	5.50
A/mallard/TN/11464/1985	H1N1	3	8	6.25
A/mallard/MN/AI07-3140/2007	H1N1	3	8	6.50
A/canvasback/ALB/276/2005	H1N1	3	8	6.25
A/blue-winged teal/ALB/212/1984	H1N1	3	8	5.75
A/mallard/MN/Sg-00628/2008	H1N1	3	8	5.75
A/shorebird/DE/318/2009	H1N1	2	9	6.50
A/mallard/MN/Sg-00627/2008	H1N1	2	10	4.94
A/shorebird/DE/274/2009	H1N1	2	7	7.25
A/green-winged teal/LA/Sg-00090/2007	H1N1	1	7	UD
A/CA/04/2009	(pdm H1N1)	4	5	6.25
A/TN/1-560/2009	(pdm H1N1)	4	5	6.50

a Lungs were collected from mice after natural death or euthanasia upon 25% weight loss, according to our protocol.

**Abbreviations:** ND, not determined; UD, undetected.

Fresh feces were collected from each group of mice on 3 and 4 dpi to examine whether viral shedding occurred via the fecal route. Viruses were isolated from most of the fecal samples tested (22/31) on 1 or both days. The hemagglutination (HA) titer and EID_50_ were determined for each fecal specimen. Although some viruses had high HA titers, the EID_50_ values were 10^1^ or lower. The highest EID_50_ detected was 10^2.25^/mL on 4 dpi from the group of mice infected with A/shorebird/DE/300/2009. This result was consistent with the intestinal titer of this group, which was 10^4.25^/mL EID_50_ on 5 dpi. In mice challenged with either of the 2009 pandemic H1N1 strains, virus was detected in the feces (10^1^/mL EID_50_) but only on 1 dpi.

### Pathogenicity of Selected Viruses in BALB/cJ Mice

**Figure 2:**
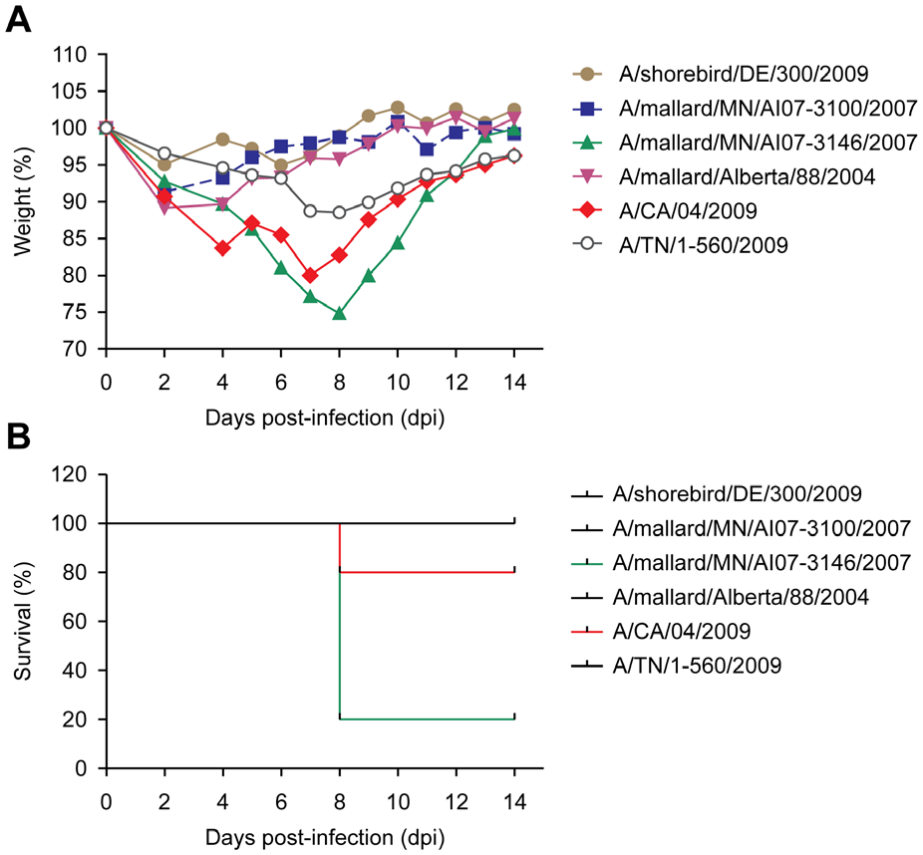
Percent weight loss and survival in BALB/cJ mice infected with 10^6^ EID_50_ of selected H1N1 isolates. (A) Daily weight loss was calculated as the percentage of weight relative to that at 0 dpi, and mean weight loss for each group is shown. (B) Mortality was recorded based on actual death or euthanasia at 25% weight loss, according to our protocol. For each virus isolate, 5 mice were infected.

BALB/cJ mice are frequently used in virologic studies and are more resistant to influenza infection than are DBA/2J mice [12]. Thus, to assess the replication efficiency and disease potential of avian H1N1 influenza viruses in BALB/cJ mice, we selected 4 PI-4 viruses and the 2 pandemic strains for screening. All mice survived infection with the pandemic strain A/TN/1-560/2009, and only 20% of those infected with A/CA/04/2009 died by 8 dpi. Among the groups of mice infected with the avian H1N1 isolates, 80% of those infected with A/mallard/MN/AI07-3136/2007 died by 8 dpi and showed a substantial weight loss (Figure 2A–B). The A/shorebird/DE/300/2009 isolate caused continuous weight loss in 40% of the infected mice by 5 dpi and in 80% by 6 dpi, though no mortality was recorded. All mice with disease symptoms in this group started to recover by 7 dpi. The A/mallard/Alberta/88/2004 isolate caused weight loss in 40% of mice by 4 dpi; however, all mice started to recover by 5 dpi. The A/mallard/MN/AI07-3100/2007 isolate caused no signs of disease during the 14 days of monitoring. These results confirmed that some avian H1N1viruses are more virulent than the 2009 pandemic H1N1 strains, not only in a mouse strain prone to influenza A infection but also in a strain that is resistant.

To better understand the efficiency of viral replication in the organs of BALB/cJ mice, we measured viral titers in the lungs and intestines. In the BALB/cJ mouse that died 8 days after being infected with A/CA/04/2009, the viral titer in the lung was less than 10^4.5^/mL EID_50_, while none was detected in the intestines. The highest viral titer in the feces of the mice infected with the pandemic A/CA/04/2009 strain was 10^2.25^/mL EID_50_ on 4 dpi; none was detected in the feces of the group infected with A/TN/1-560/2009. The viral titer in the lungs was determined only for mice infected with A/mallard/MN/AI07-3136/2007; because the other groups did not show considerable disease symptoms, they were simply monitored for weight loss. The average lung titer was 10^6.5^/mL EID_50_ on 6 dpi, while the average intestinal titer was 10^2.5^/mL EID_50_. The viral titers detected in the lungs and intestines of BALB/cJ mice were comparable to those detected in the same tissues of DBA/2J mice on the respective days. The feces collected from each group were also tested for viral titers on 3 and 4 dpi. The titers varied from 10^1^/mL to 10^3.5^/mL EID_50_.

**Figure 3:**
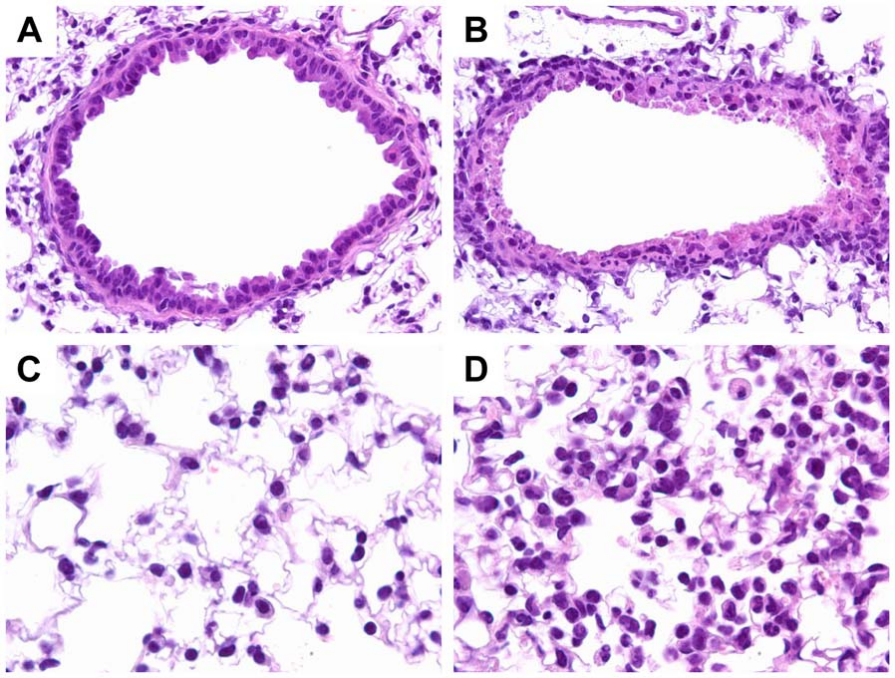
Hematoxylin & eosin staining of influenza-infected lungs from DBA/2J mice. (A) In the uninfected lung, the bronchiole is lined with epithelium. (B) The infected lung shows severe bronchiole epithelial necrosis. (C) In the uninfected lung, the alveoli lumens are free of cells and have thin septal walls. (D) The infected lung shows focal alveolitis with neutrophils and mononuclear cells in the alveoli lumens, and the alveolar septal walls are thickened. Magnification: 40×(A–B) or 80×(C–D).

The lungs of the DBA/2J mice and BALB/c mice had similar pathology, but the lesions in the former strain were slightly more extensive and severe than those in the latter. The lungs had necrotizing bronchitis and bronchiolitis with acute patchy alveolitis in the adjacent alveoli (Figure 3). Pathologic changes were not observed in any of the other organs examined.

### Pathogenicity and Transmission in Ferrets

**Figure 4:**
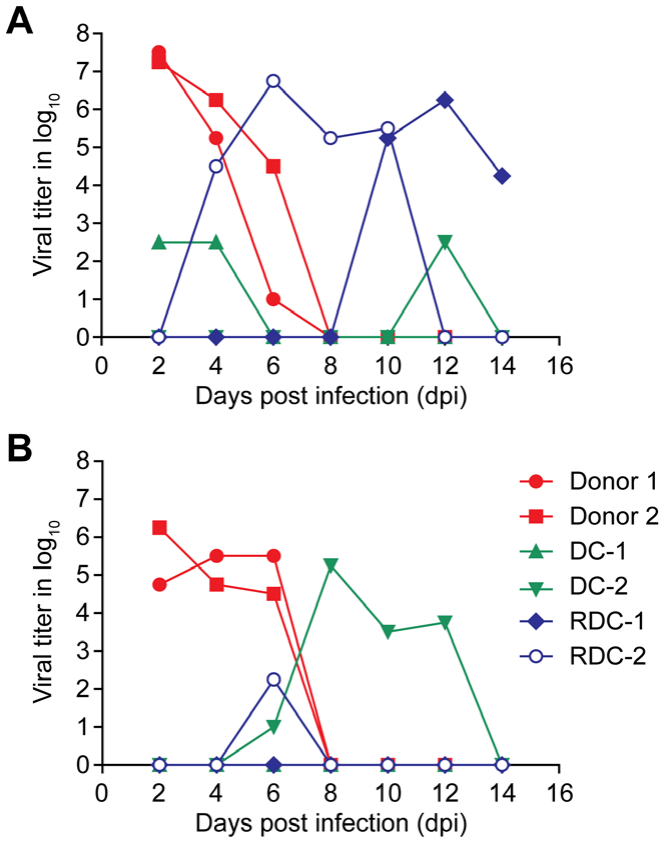
Transmissibility of A/shorebird/DE/300/2009 in ferrets. Viral titers were detected in (A) nasal washes and (B) rectal swabs of donor ferrets (red), direct-contact ferrets (DC, green) and respiratory-droplet–contact ferrets (RDC, blue) 14 dpi. Data from each animal are shown. Viral titers are given in terms of EID_50_.

Although the mouse model is widely used in influenza research, ferret model more closely mimics humans, in terms of their responses to influenza infection. Therefore, understanding disease progression, pathogenicity, and transmissibility of avian H1N1 viruses in the ferret model is essential. Two representative viruses, A/shorebird/DE/300/2009 from the PI-4 category and A/mallard/MN/Sg-00627/2008 from PI-2, were assessed to determine their pathogenicity and transmissibility via direct or respiratory-droplet transmission. Both viruses caused disease signs in the donor ferrets (i.e., those that were directly infected). The donor ferrets inoculated with each virus showed fever, sneezing, and reduced bright-alert response at varying degrees; however, no significant weight loss was observed in any donor ferret. Diarrhea was observed in 1 donor ferret infected with A/shorebird/DE/300/2009. High viral titers were detected in the nasal washes of all donor ferrets infected with either virus on 2 to 6 dpi (Figure 4A). However, only those infected with A/shorebird/DE/300/2009 showed high viral titers in the rectal swabs (Figure 4B). The viral titers were almost undetectable in the rectal swabs of the ferrets infected with A/mallard/MN/Sg-00627/2008; only 1 donor ferret shed 10^1^/mL EID_50_ virus on 6 dpi in the rectal swab.

Transmission by direct contact was indicated by the nasal washes for each virus tested. However, viral shedding in the direct-contact ferrets was detected on different days (Figure 4A). The transmission of the virus via respiratory droplets was observed only in the ferrets caged across from the donor ferrets infected with A/shorebird/DE/300/2009. One of the ferrets infected via respiratory-droplet transmission (RDC-2) started shedding virus in the nasal wash 4 dpi (Figure 4A), with clinical symptoms of fever, reduced bright-alert response, sneezing, and nasal discharge. The infection cleared by 12 dpi. Viral titer in rectal swabs of this ferret was detected only 6 dpi at 10^2.25^/mL EID_50_. The second ferret infected by respiratory-droplet contact (RDC-1) started shedding the virus in nasal washes 10 dpi and developed clinical symptoms 13 dpi similar to those of the other ferret infected via respiratory-droplet contact. The viral titer in nasal wash was not detected after 14 dpi, and that in rectal swabs was undetectable during the study period. Compared to ferrets inoculated with the low-pathogenic A/mallard/MN/Sg-00627/2008, ferrets inoculated with the A/shorebird/DE/300/2009 isolate (passaged in eggs twice), which was previously characterized as one of the most pathogenic of the viruses tested in DBA/2J mice, showed a higher rate of infection with higher viral titers both in nasal washes and rectal swabs and successful transmission through direct or respiratory-droplet contact.

### Pathogenicity of Other Subtypes of Low-Pathogenic avian Influenza a Viruses in DBA/2J Mice

To determine whether other subtypes of avian influenza A viruses are as pathogenic as avian H1N1 viruses in DBA/2J mice, we screened 18 strains of influenza A viruses from 6 other hemagglutinin (HA) subtypes. Three virus isolates of different HA subtypes were grouped as moderately pathogenic (PI-3). A/shorebird/DE/124/2001 (H6N2) and A/shorebird/DE/260/2000 (H10N4) isolates caused 100% mortality, but A/mallard/NY/6750/1978 (H2N2) caused 80% mortality (Table 4). Four virus isolates [A/ruddy turnstone/DE/79/1999 (H3N2), A/mallard/ALB/35/2001 (H4N6), A/shorebird/DE/321/2009 (H10N1), and A/mallard/ALB/182/2007 (H10N7)] were categorized as PI-2, and A/blue-winged teal/ALB/604/1978 (H2N2) was categorized as PI-1 based on their pathogenicity scores. Among all subtypes of avian viruses tested, at least 1 isolate of each HA subtype caused morbidity and mortality in DBA/2J mice, while none of the H12 isolates caused disease signs. Ten of the virus isolates were nonpathogenic (PI-0) in DBA/2J mice.

**Table 4:** Percent mortality and pathogenicity in DBA/2J mice infected with various subtypes of avian influenza A viruses (at 10^6^ EID_50_).

Influenza A virus isolate	Subtype	Mortality (%)	Survival score^a^	Weight loss score^b^	Total pathogenicity score^c^	Pathogenicity Index^d^
A/duck/NJ/7872-27/1995	H2N2	0	0.800	0.207	1.007	0
A/blue-winged teal/ALB/604/1978	H2N2	20	0.720	0.194	0.914	1
A/mallard/NY/6750/1978	H2N2	80	0.389	0.176	0.565	3
A/ruddy turnstone/DE/79/1999	H3N2	60	0.571	0.186	0.758	2
A/ruddy turnstone/DE/81/1994	H3N2	0	0.800	0.206	1.006	0
A/ruddy turnstone/DE/230/1999	H3N2	0	0.800	0.199	0.999	0
A/mallard/ALB/121/2008	H4N6	0	0.800	0.201	1.001	0
A/mallard/ALB/35/2001	H4N6	40	0.583	0.207	0.790	2
A/shorebird/DE/309/2008	H4N6	0	0.800	0.196	0.996	0
A/ruddy turnstone/DE/293/2006	H6N2	0	0.800	0.188	0.988	0
A/shorebird/DE/124/2001	H6N2	100	0.400	0.088	0.488	3
A/shorebird/DE/707/2009	H6N2	0	0.800	0.198	0.998	0
A/shorebird/DE/321/2009	H10N1	80	0.549	0.181	0.730	2
A/shorebird/DE/260/2000	H10N4	100	0.400	0.088	0.488	3
A/mallard/ALB/182/2007	H10N7	75	0.614	0.197	0.811	2
A/shorebird/DE/43/2007	H12N1	0	0.800	0.216	1.016	0
A/shorebird/DE/488/2008	H12N3	0	0.800	0.216	1.016	0
A/ruddy turnstone/DE/107/2007	H12N5	0	0.800	0.217	1.017	0

a The survival score was calculated as 0.8× (survival AUC/maximum AUC).

b The weight loss score was calculated as 0.2× (weight loss AUC/maximum AUC).

c The total pathogenicity score is the sum of the survival and weight loss scores.

d Pathogenicity indexes were classified as follows: 3, moderately pathogenic; 2, low pathogenic;1, least pathogenic; and 0, nonpathogenic.

## Discussion

In recorded human history, H1N1 influenza viruses have been a major cause of human pandemics. Because these viruses have successfully established themselves in the human, swine, and avian populations, there is substantial risk of the gene pools of viruses from different host origins mixing. An influenza A pandemic occurred in 2009, and its genetic constellation of human, swine, and avian origin [9] again highlighted the potential risk of H1N1 viruses and the importance of the gene pool in the wild bird population. In this study, we determined the pathogenicity of a group of representative avian H1N1 viruses of North American lineage in the mouse model. We further evaluated a few selected viruses in a ferret model to determine the pathogenicity and transmissibility of avian H1N1 viruses.

Although the severity of disease caused by the H1N1 viruses varied in the mouse model, 10 of 31 isolates were placed in the most pathogenic (PI-4) category. Of those 10 viruses, 8 were as pathogenic or more pathogenic than the pandemic A/TN/1-560/2009 virus, while the pathogenicity scores of 2 isolates were almost identical to that of the pandemic A/CA/04/2009 virus in DBA/2J mice. Thus, some avian H1N1 viruses are more pathogenic than the 2009 pandemic strains in the DBA/2J mouse model.

The pandemic A/CA/04/2009 virus requires as many as 9 lung-to-lung passages to be lethal in BALB/cJ mice [14]. The 2 pandemic strains used in this study were also much less pathogenic (PI-0 to PI-1) in BALB/cJ mice. Considering BALB/cJ mice are more resistant to influenza A infection than are DBA/2J mice, the fact that the A/mallard/MN/AI07-3136/2007 isolate with 80% morbidity was more pathogenic than A/CA/04/2009 or A/TN/1-560/2009 in BALB/cJ mice without mouse adaptation raises concern about the avian H1N1 viruses residing in wild bird populations. Overall, our findings indicate that some avian H1N1 viruses in the wild bird reservoir could be more virulent than the 2009 pandemic strains in mammals and could be a potential concern for veterinary and human public health.

The main goal of this study was to evaluate the disease potential of low-pathogenic avian H1N1 influenza viruses in mammalian models, but we also tested other subtypes of low-pathogenic avian influenza A viruses to compare their pathogenicity levels. We selected the viruses from different categories: the ones detected in humans (H2, H3), those of avian origin but previously detected in mammals (H4, H6, H10), and those of purely avian origin and not yet detected in mammals (H12). In many instances, the DBA/2J mice either did not show any symptoms or recovered from the disease within a few days. The H10N4 and H10N7 isolates that caused mortality in DBA/2J mice were previously characterized in minks [15]. Therefore, the higher pathogenicity of these viruses in a mouse model can be possibly explained by the tendency of H10N4 and H10N7 viruses to cross the species barrier under certain circumstances. H6 viruses are infectious to mice and ferrets [16]. We found that 1 isolate, A/shorebird/DE/124/2001 (H6N2), was pathogenic in the mouse model. Likewise, H4N6 viruses have been isolated from pigs [17]. Our results confirm the potential pathogenicity of H4N6 avian influenza A viruses in mammals. None of the H12 isolates used in this study caused disease signs in the mouse model, thereby confirming the absence of H12 viruses in mammals so far. Although several isolates were variably virulent in the DBA/2J mice, none were characterized in the PI-4 category. These findings suggest that H1N1 influenza A viruses of avian origin are prone to be more pathogenic in mammals than are many other avian influenza A viruses.

The pathogenicity and transmissibility of the 2009 pandemic H1N1 viruses via direct or respiratory-droplet contact was previously reported in a ferret model [18], [19]. Our results indicate that 2 avian H1N1 viruses used in this study caused infection in donor ferrets, as well as in ferrets in direct contact with those donors. Furthermore, the A/shorebird/DE/300/2009 isolate successfully transmitted to respiratory-droplet naïve-contact ferrets, as was seen for the pandemic 2009 H1N1 strains.

The ferret model is the best mammalian model widely used in influenza research, because it closely mimics the progression, pathogenicity, and transmissibility of influenza A infections in humans. However, this animal model is high maintenance, i.e., ferrets are more expensive; they require more space per animal, and their care is labor-intensive. These factors are a big obstacle for a broad experimental setup to assess large numbers of avian viruses. Although mice are not naturally infected with influenza viruses, and inbred strains lack the Mx gene to inhibit viral replication [20], [21], mice are still widely used because they are a low-maintenance animal model (i.e., more reagents and serum are available for vaccine and antiviral assays; mice also cost less, require less space per animal, and are less labor-intensive). Although BALB/cJ mice have been a broadly preferred model, recent studies have shown that DBA/2J mice are more sensitive to influenza A infection than BALB/cJ [12] or C57BL/6 mice [13]. High virulence of avian H1N1 viruses in the DBA/2J confirms that this strain is a suitable model in which to conduct initial pathogenicity screens of large numbers of viruses. Moreover, varying degrees of pathogenicity and transmissibility of 2 selected avian H1N1 viruses from 2 separate pathogenicity levels in a ferret model confirm the PI values assigned in the DBA/2J model. Further assessment of several more avian H1N1 viruses from the PI-4 category and the representative virus isolates from other pathogenicity levels in the ferret model would support DBA/2J mice as a suitable mammalian model for the initial screening of vast quantities of avian H1N1 viruses from the wild bird repository. This is an important first step in establishing an efficient, long-term risk-assessment and risk-management program for H1N1 influenza A viruses of avian origin.

Influenza A viruses have been previously detected in the intestines of ferrets and pigs [22]. The viral titers we detected in the intestines of some mice on 5 dpi were consistent with those earlier findings. During the 2009 pandemic, the virus spread fast and was a global threat due to modern transportation routes. Many people were hospitalized with severe clinical symptoms. Although influenza A generally causes upper respiratory tract illnesses in humans, the 2009 pandemic caused lower respiratory tract infections, as well as gastrointestinal symptoms (i.e., nausea, vomiting, and diarrhea), especially in children and healthy young adults [23]. Our findings of virus isolates in the feces of mice challenged with most of the avian H1N1 viruses and diarrhea in some animals were consistent with the findings from the stool samples of patients during the 2009 pandemic [24]. Observation of diarrhea in our study and high viral titers in the rectal swabs of ferrets infected with avian H1N1 virus raise concern about the potential shedding of the viruses in the feces and possible viral replication in the intestines. We still do not know whether the virus replicates in the intestines and is shed via the fecal route, or if it replicates only in the upper and lower respiratory tract and is simply displaced into the gastrointestinal tract via mucus secretion. Molecular analyses and detailed histopathologic examination are necessary to definitively answer this question.

This study serves as an initiative for a broader assessment. Here we have presented only the biological assessments of avian H1N1 viruses; further confirmation using molecular approaches such as genotyping and mutation analyses are needed. The genetic constellations of these viruses, the evolutionary distances among them, and the molecular changes in the virus genome that are associated with pathogenesis (i.e., potential pathogenicity markers), are areas that require further study. The implementation of a risk-assessment and risk-management program for avian H1N1 viruses worldwide would better estimate what to expect from nature and thus allow influenza researchers to be prepared to preserve public health.

The frequent circulation of H1N1 influenza A viruses in the human population and the potential pandemic risk that these viruses pose emphasize the necessity for a risk-assessment and risk-management program for H1N1 viruses residing in the wild bird population. The first step toward developing such a program would be the selection of a suitable small animal model in which to screen a large number of viruses to determine their potential pathogenicity and to facilitate a rapid response when warranted. As can be seen from our results, some avian H1N1 viruses residing in the natural reservoir can adapt and make the transition smoothly from birds to mammals, even without using an intermediate species. In fact, our findings in ferrets raise concern about the avian H1N1 viruses in terms of public health. More efficient surveillance studies and an efficient risk-assessment program for avian H1N1 viruses in nature are needed to identify the virulence levels by screening as many virus isolates as possible. Here we examined only the pathogenicity of avian H1N1 viruses of North American lineages, but pathogenicity is only one aspect of risk assessment. Therefore, a broader surveillance program would be indispensable for more efficient risk-assessment and risk-management of these and other influenza viruses. The significance of H1N1 viruses in wild bird populations and the vast diversity in that gene pool should be highlighted, because those viruses have the potential to bring a new pandemic strain into existence, either via direct transmission or mixing with other viruses that originate in a different host.

## Materials and Methods

### Ethics Statement

All animal experiments were conducted in an Animal Biosafety Level 2+ (level 2 with enhanced biocontainment for pandemic H1N1 influenza A virus) facility at St. Jude Children’s Research Hospital, in compliance with the policies of the National Institutes of Health and the Animal Welfare Act and with the approval of the St. Jude Children’s Research Hospital Institutional Animal Care and Use Committee (Protocol Number: 081, approval date: Aug. 19, 2011).

### Viruses

Thirty-one isolates of avian H1N1 viruses were selected for this study from the North American avian influenza A repository at St. Jude Children’s Research Hospital and at the Department of Population Health, College of Veterinary Medicine, University of Georgia, Athens, GA. A/mallard/OH/4809-9/2008 and A/northern shoveler/MO/466554-14/2007 were kindly provided by Dennis Senne at the United States Department of Agriculture National Veterinary Services Laboratories, Diagnostic Virology Section, USDA-APHIS, Ames, IA; Tom DeLiberto and Seth Swafford at the United States Department of Agriculture Animal and Plant Health Inspection Service, Wildlife Services, National Wildlife Disease Program, Fort Collins, CO. The principal criterion was that the viruses were H1N1 reproduction of the time intervals from 1984 to 2009. The viruses were selected from our active surveillance study, as well as to follow up to NA inhibitor study [25] for further characterization. Six of H1N1 viruses were isolated before 2000, and 25 were isolated after 2000 (Table 1). We included two 2009 pandemic H1N1 isolates (A/CA/04/2009 and A/TN/1-560/2009) to make a comparison. The 31 representative viruses and the 2 pandemic strains were minimally propagated (passage 2, 3, or 4) in the allantoic cavities of 10- to 11-day-old chicken eggs to avoid further molecular variation. The EID_50_ was determined for each virus stock by inoculating the chicken eggs with 10-fold serial dilutions of the viruses. All virus dilutions were prepared in a solution of sterile PBS and antibiotics. EID_50_ values were calculated by the Reed-Muench method [26].

### Animals

Six-week-old female mice with the DBA/2J or BALB/cJ genetic background were purchased from the Jackson Laboratories (Bar Harbor, ME). Upon arrival, all mice were acclimatized in the Animal Resources Center at St. Jude for 1 week before infection, and they were given food and water ad libitum.

Three-to 4-month-old outbred male ferrets were purchased from Triple F Farms (Sayre, PA). Upon arrival, all ferrets were quarantined in the Animal Resources Center at St. Jude for 1 week before virus inoculation and were given food and water ad libitum. The ferrets used in this study were confirmed influenza-seronegative by hemagglutination inhibition assay, i.e., their hemagglutination inhibition titers were less than 10 against A/Perth/16/2009 (H3N2) and A/CA/04/2009 (H1N1) viruses.

### Pathogenicity Screening in DBA/2J Mice

All 31 avian H1N1 isolates and the two 2009 pandemic H1N1 strains (A/CA/04/2009 and A/TN/1-560/2009) were screened for pathogenicity in DBA/2J mice. Virus dilutions were prepared in sterile PBS to obtain 10^6^ infectious particles, and 30 µL was used to infect each mouse intranasally. A group of 5 DBA/2J mice were inoculated with each virus after being anesthetized with avertin (2,2,2-tribromoethanol; Acros Organics; Morris Plains, NJ). Upon virus challenge, mice were monitored for 14 dpi for disease symptoms (scruffy fur, hunched appearance, and reduced bright-alert response), weight loss, and mortality. Mortality was recorded based on actual death or a 25% weight loss cut-off, according to our animal protocol.

The total pathogenicity score was calculated as the weighted sum of percentage of survival by time and percentage of weight loss by time scores. The mortality rate was considered more important than weight loss, especially for the initial screening. Therefore, in the weighted sum, 80% weight was given to survival and 20% to weight loss. Based on the total pathogenicity scores, viruses were divided into 5 categories and assigned a PI value of 0 through 4, with 0 being nonpathogenic and 4 being the most pathogenic.

Statistical analyses were done by one-way ANOVA with Bonferroni’s multiple comparison test to compare each group of viruses with the others and a one-sample *t*-test to compare the average pathogenicity score of each group to that of individual pandemic strains using GraphPad Prism version 5 for Windows (GraphPad Software Inc., La Jolla, CA). Differences were considered statistically significant at P<0.05.

In addition to avian H1N1 viruses, 18 avian influenza A isolates belonging to 6 other HA subtypes (H2N2, H3N2, H4N6, H6N2, H10, and H12) were selected for pathogenicity screening in DBA/2J mice (Table 4). Groups of 5 DBA/2J mice were challenged intranasally with 10^6^ infectious particles after anesthesia by avertin, as mentioned previously. Each group was monitored for 14 dpi, and weight loss and mortality were recorded daily.

### MLD_50_ Determination

The MLD_50_ was determined for selected groups of viruses from 3 pathogenicity levels. PI-4 viruses included A/mallard/ALB/119/1998, A/mallard/ALB/201/1998, A/shorebird/DE/300/2009, A/mallard/MN/AI07-3136/2007, A/mallard/ALB/88/2004, A/mallard/MN/AI07-3100/2007, and A/shorebird/DE/324/2009; PI-3, A/blue-winged teal/LA/B228/1986; and PI-2, A/mallard/MN/Sg-00627/2008. Ten-fold serial dilutions were prepared in a solution of sterile PBS and antibiotics from 10^6^ EID_50_ to 10^1^ EID_50_. Infections in mice were performed as mentioned previously. MLD_50_ values were calculated in terms of EID_50_ by the Reed-Muench method [26].

### Pathogenicity in BALB/cJ Mice

Four avian H1N1 viruses from the PI-4 category (A/shorebird/DE/300/2009, A/mallard/MN/AI07-3100/2007, A/mallard/MN/AI07-3136/2007, and A/mallard/Alberta/88/2004) were selected for further screening in BALB/cJ mice. Infection was performed as mentioned previously using 10^6^ infectious particles of each virus, and each mouse was challenged intranasally with 30 µL of virus suspension.

### Viral Titers in Lungs and Intestines

Mice were euthanized when they reached 25% weight loss, and lungs and intestines were collected to determine the viral titers. Additional organs, including spleen, liver, brain, and heart were collected for assay. All organs were homogenized in sterile PBS using the Qiagen Tissue Lyser II (Qiagen, Gaithersburg, MD). Organ homogenates were centrifuged at 2000×*g* for 5 min, and the supernatants were transferred to clean tubes from which 10-fold dilutions (10^−1^ to 10^−10^) were made in a solution of sterile PBS and antibiotics. For each dilution, three 10- to 11-day-old chicken embryos were inoculated through the allantoic cavity to determine the viral titers for each organ in terms of EID_50_.

### Histopathology

Tissue for histological examination was collected from all organs and fixed in 10% neutral buffered formalin. The fixed tissue was embedded in paraffin, sectioned at 4-µm, stained with hematoxylin and eosin, and examined by light microscopy in a blinded fashion.

### Determination of Viral Titers in Feces

During the pathogenicity screening with 10^6^ EID_50_, fresh feces were collected from each group of mice 3 and 4 dpi and resuspended in sterile PBS. All feces specimens were collected directly from mice, avoiding any contact with the cage. The viral titers in the feces were determined in terms of EID_50_ on the day of fecal sample collection. The EID_50_ for each specimen was determined by inoculating the allantoic cavities of chicken eggs with 10-fold serial dilutions of the fecal suspension (undiluted through 10^−5^) in sterile PBS and antibiotics; hemagglutination activity was determined for the viruses isolated from the allantoic fluid of chicken eggs inoculated with undiluted fecal suspension and antibiotics.

### Pathogenicity and Transmission in Ferrets

The donor ferrets were slightly anesthetized with isoflurane and inoculated intranasally with 0.5 mL 10^6^ EID_50_ of A/shorebird/DE/300/2009 or A/mallard/MN/Sg-00627/2008 (representative of PI-4 and PI-2 category viruses, respectively). For the transmission study, 1 direct-contact ferret was placed in the cage with a donor ferret on 0 dpi. One respiratory-droplet–contact ferret was placed in a different cage that was separated from the donor and direct-contact ferrets by a grill divider [18]. This setup was duplicated for each virus (n = 6 ferrets per virus). Following the inoculation of donor ferrets, each ferret was weighed; their temperature was measured; and clinical signs (i.e., weight loss, sneezing, nasal discharge, diarrhea, and reduced bright-alert response) were monitored every other day. In addition, nasal wash specimens were collected in 1-mL sterile PBS under light anesthesia with ketamine (40 mg/kg), and rectal swabs were collected in the isolation media (50% glycerol in PBS containing 1000 units/mL penicillin, 200 µg/mL streptomycin, 50 units/mL nystatin, 250 µg/mL gentamicin, and 100 units/mL polymyxin B).

Nasal wash and rectal swab specimens were serially diluted in sterile PBS with antibiotics and inoculated into 10- to 11-day-old chicken eggs to determine the viral titers in terms of EID_50_, as mentioned previously.
